# Are people consistent when reading nonwords aloud on different occasions?

**DOI:** 10.3758/s13423-021-01925-w

**Published:** 2021-05-13

**Authors:** Anastasia Ulicheva, Max Coltheart, Oxana Grosseck, Kathleen Rastle

**Affiliations:** 1 Department of Psychology, Royal Holloway, University of London, Egham, TW20 0EX UK; 2 Department of Cognitive Science, Macquarie University, Sydney, Australia

**Keywords:** Nonword reading, Reading aloud, Variability, Individual differences, Reading experience, ﻿Spelling–sound

## Abstract

Tests of nonword reading have been instrumental in adjudicating between theories of reading and in assessing individuals’ reading skill in educational and clinical practice. It is generally assumed that the way in which readers pronounce nonwords reflects their long-term knowledge of spelling–sound correspondences that exist in the writing system. The present study found considerable variability in how the same adults read the same 50 nonwords across five sessions. This variability was not all random: Nonwords that consisted of graphemes that had multiple possible pronunciations in English elicited more intraparticipant variation. Furthermore, over time, shifts in participants’ responses occurred such that some pronunciations became used more frequently, while others were pruned. We discuss possible mechanisms by which session-to-session variability arises and implications that our findings have for interpreting snapshot-based studies of nonword reading. We argue that it is essential to understand mechanisms underpinning this session-to-session variability in order to interpret differences across individuals in how they read nonwords aloud on a single occasion.

In order to read aloud a nonword such as BAMPER, the reader must utilise their stored knowledge of the relationship between letters (e.g., *M*) and sounds (/m/), as well as larger units (e.g., ER–/ə/). Nonword reading tasks have been used widely to probe readers’ knowledge of letter–sound relationships and how they exploit this knowledge. These tests are used in schools for assessing the effectiveness of reading instruction (Castles et al., 2018), and they are used in clinical settings to diagnose reading impairment (Coltheart, 2006; Rack et al., 1992). Finally, researchers use nonword reading data to adjudicate between theories and models of reading (Andrews & Scarratt, 1998; Coltheart et al., 2001; Mousikou et al., 2017; Perry et al., 2007; Plaut et al., 1996; Pritchard et al., 2012).

Large-scale studies have highlighted the fact that nonword reading in English is variable (Mousikou et al., 2017; Pritchard et al., 2012). That is, different people may produce different responses to a single nonword. On one hand, this variability poses a challenge for existing theories and models of reading, because it calls into question the concept of the “average” reader that the models aim to simulate. On the other hand, this variability can be an asset for the study of individual differences. This is because individual’s knowledge may be shaped by their reading experience (Steacy et al., 2019)—that is, the type and the quantity of texts they encounter.

One example that illustrates how inferences of this type are drawn comes from a computational study by Zevin and Seidenberg (2006). The authors implemented multiple versions of a parallel distributed processing model that learned to read. These models were trained using different sets of words, simulating differences in reading experience. The different models came to read nonwords in different ways. Zevin and Seidenberg’s simulations provide a preliminary answer as to why nonword reading varies across individual readers: Their reading experience is different. However, we cannot interpret differences across readers unless we can confirm that nonword reading tests adequately reflect the stable spelling–sound knowledge of an individual.

The goal of the present study is to determine whether adults’ nonword reading aloud responses are identical from one testing session to the next. High intersession consistency would suggest that nonword reading responses reflect a stable body of spelling–sound knowledge. In contrast, low intersession consistency would raise a series of further questions about why variability arises and its implications for making inferences on the basis of a single snapshot of performance. We posited two candidate factors that might constrain intersession variability: spelling–sound consistency (an item-based factor) and literacy skill as a proxy for reading experience (a participant-based factor). We reasoned that if a stochastic component were added to units within a model of reading (e.g., Rueckl et al., 2019), then the impacts would be greatest on reading aloud those graphemes with multiple possible pronunciations (e.g., EA pronounced as short or long, /ɛ/ as in BREAD or /iː/ as in BREATHE). Likewise, these impacts should be greatest on readers with a high degree of literacy skill likely to have greater knowledge of the multiple pronunciations associated with particular graphemes (e.g., due to their experience with rare words and loan words; see Siegelman et al., 2020; Steacy et al., 2019; Treiman & Kessler, 2006). This rationale led us to predict that intersession variability might be greatest for nonwords comprising graphemes with many possible pronunciations and for participants with a higher degree of reading experience. Our results prompted further questions regarding the dynamics of session-to-session variation, which we explored in two post hoc analyses.

## Methods

### Participants

Participants were 27 undergraduate students at Royal Holloway, University of London. Testing took place between November 2019 and March 2020, with each participant attending five sessions. Data from five participants were removed due to technical issues or because the participants dropped out of the study. Two further participants of the remaining 22 completed only four sessions; testing was interrupted due to the coronavirus pandemic. Their data were retained. Participants were, on average, 21 years old (range was 19–28 years; four males and 18 females). All described themselves as native English speakers with no history of reading, spelling, or learning difficulties. Participants were paid for their time.

### Materials

Fifty nonwords were selected from a megastudy of disyllabic nonword reading (Mousikou et al., 2017). In order to ensure that the selected nonwords varied in terms of how much variability they elicited across participants in the original study, we drew random samples repeatedly until a normal distribution was obtained (see Appendix). Five practice items were also selected randomly from the same study.

Literacy skill was estimated using spelling and vocabulary tests. Vocabulary knowledge was assessed using the corresponding subscale of the Shipley Institute of Living Scale (Shipley, 1940). The test required participants to select the most appropriate meaning out of four alternatives and included 40 printed words of increasing difficulty. The spelling test required participants to type the spellings of 40 words adapted from Burt and Tate (2002). Each word was played through headphones, first in isolation and then in a sentence context.

#### Measures

##### Item consistency

This was a continuous measure that characterised a nonword’s graphemes in terms of how predictable their pronunciations are in real English words. First, we measured the certainty with which graphemes were associated with their pronunciation(s) in a corpus of existing English words. Then, we applied these measures to graphemes in our nonwords. We reasoned that if a grapheme corresponds to multiple pronunciations in the English writing system, then nonwords comprising such graphemes would be read more variably.

Real-word grapheme-to-phoneme statistics were obtained in the following way. We considered monosyllabic and polysyllabic words that most English speakers know (i.e., prevalent words; Brysbaert et al., 2019). Phonemic transcriptions for these words were obtained from the CELEX database (Baayen et al., 1993). We parsed individual syllables^1^ in CELEX words into graphemes using the parsing algorithms implemented in the DRC model (Coltheart et al., 2001). Only syllables where the number of parsed graphemes was equal to the number of phonemes were analysed (433,833 syllables, or 37,019 unique words), so that each grapheme could be unambiguously put into correspondence with its phoneme. Some within-syllable contextual information was retained. This information was related to the position of graphemes within syllables and in specific cases, surrounding graphemes (as indicated by the DRC model). For example, grapheme I tends to sound as /I/ in word beginnings (e.g., ILLUSION), whereas it receives more varied pronunciations word-finally (e.g., /aɪ/ as in FUNGI, and /i/ as in KIWI). Further, graphemes’ pronunciations often depend on the surrounding context. For example, grapheme C’s most frequent pronunciation is /k/ (ACORN); however, when it precedes vowels, such as E, I, Y, the pronunciation is more likely to be /s/ (MERCY). Using this approach, we extracted 500 context-dependent grapheme-to-phoneme correspondences from CELEX along with their relative frequencies (i.e., the frequency of grapheme-to-phoneme correspondence divided by the frequency of this grapheme). Next, we calculated entropy of possible pronunciations for each grapheme in the real-word corpus using the formula Σ[–*p*_*i*_ × ln(*p*_*i*_)] (Shannon, 1948), where *p*_*i*_ is relative frequency of each grapheme-to-phoneme correspondence for this grapheme.

These real-word grapheme entropy values were applied to our nonwords in the following way. Nonwords were manually parsed into syllables. and grapheme parsings were obtained for these, as was done with real words. We calculated the several metrics of nonword-level consistency by averaging or adding entropy values for individual graphemes and then syllables, or selecting the highest value within each unit (see the corresponding R script for details).^2^ The consistency value that had the highest correlation with the dependent variables (DVs) used in this paper was entropy of the most inconsistent syllable within the nonword (where grapheme entropies within each syllable were averaged), so we chose to use this metric (see the R script for details). The metric was multiplied by –1 so that low consistency values indicated that the nonword includes graphemes that have unpredictable pronunciations (ARROSTE has item consistency of –1.05), whereas high consistency values indicate that the nonword consists of graphemes whose pronunciations are predictable (BLISPLE’s item consistency is –0.21). In what follows, we will refer to this measure as “item consistency” for simplicity. The variable was logarithm-transformed and then centred.

##### Literacy skill

Spelling and vocabulary measures were correlated with each other (*r* = .5, *N* = 21, *p* = .021). We therefore designed a composite measure for use in the statistical model to avoid multicollinearity. This composite measure was the average between normalised spelling and vocabulary scores. The variable ranged between –1.5 and 1.6, with a mean of –.01.

### Procedure

Testing took place at Royal Holloway, Department of Psychology. Each participant was required to come to the lab five times. Sessions were separated by at least 7 days (maximum, 58 days; mean, 8 days; median, 7 days). DMDX software was used for stimulus presentation and response recording (Forster & Forster, 2003). Participants were asked to read words aloud as quickly and clearly as possible.^3^ Experimental nonwords appeared in a random order, one at a time in the centre of the screen, white on black background. Stimuli were presented in a 28-point Courier New font. Each stimulus was displayed for 3,000 ms. Participants’ distance from the monitor and viewing angle were not controlled. Participants’ reading-aloud responses were recorded. The duration of each reading-aloud session was under 5 minutes. In two out of the five sessions, each participant received either the vocabulary or the spelling test in addition to the read-aloud task, which increased the duration of these sessions by up to 10 minutes. These tasks were administered using E-Prime 2.0. Time for responding was unrestricted.

## Analysis 1: Is there variability in how individuals read nonwords aloud on different occasions?

**Fig. 1:**
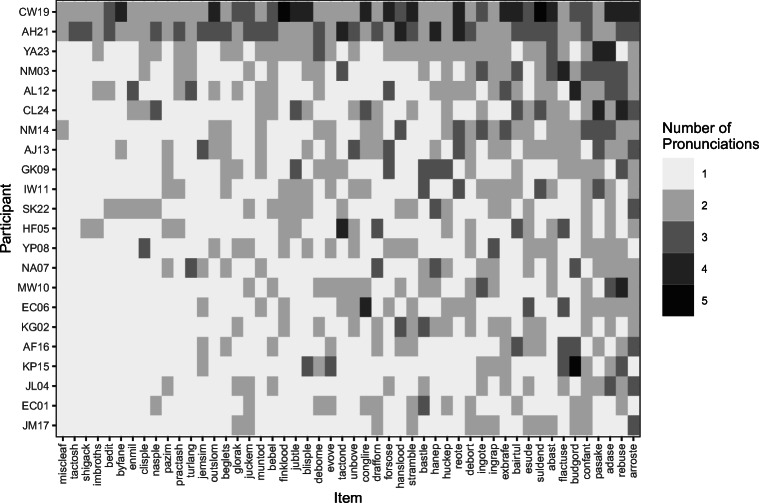
The number of different pronunciations assigned to every nonword (*x*-axis) by every participant (*y*-axis). The axes were arranged according to the average number of pronunciations that were generated across occasions, ranging from the least variable to the most variable nonword (from left to right) and participant (from bottom to top)

Reading-aloud responses were transcribed by Oxana Grosseck. We ignored information about lexical stress for simplicity. All analyses were performed in the statistical software R (Version 4.0.4; R Core Team, 2021). For each nonword and participant, we counted the number of different responses produced across sessions. This number ranged from 1 (i.e., response was the same across all occasions) to 5 (i.e., response was different on each occasion). These data are represented visually in Fig. 1. Averaged across nonwords (the columns of the matrix in Fig. 1), participants differed in their variability across sessions. Participant variation ranged from a minimum of 1.28 to a maximum of 2.86. On average, participants produced 1.61 different pronunciations to each nonword across sessions, which is significantly different from the population mean of 1, μ = 1; *t*(21) = 7.271, *p* < .001. For items, the mean number of different responses produced across sessions ranged from 1.14 to 2.27 (mean was 1.61; significantly different from μ = 1; *t*(49) = 15.841, *p* < .001. This analysis demonstrates that people do not read nonwords in the same way when tested repeatedly.

## Analysis 2: What drives variability across sessions?

Figure 1 suggests that variability that we observed across testing sessions is not random: Certain participants (such as CW19) and certain nonwords (such as ARROSTE) generate demonstrably more variability across sessions than others. We sought to assess whether this variability could be explained by an item-level factor measuring print-to-sound consistency of nonwords and a participant-level factor reflecting participants’ literacy skill (see *Materials* for details).

Our dependent variable, response diversity, captured variability within a given participant’s responses to a given nonword across all sessions. This variable was operationalised as entropy (H) for consistency with previous research (Mousikou et al., 2017). Response diversity was calculated using the formula Σ[–*p*_*i*_ × ln(*p*_*i*_)], where *p*_*i*_ is the proportion of sessions where a given nonword was pronounced in a specific way. For example, participant AF16 reading BLISPLE in the same way across all five sessions corresponds to response diversity of 0 (the minimum value). Participant KP15 pronounced BUDGORD in five different ways (response diversity is 1.6, the maximum value for five sessions).

The model of response diversity included item consistency and literacy skill, their interaction, two random intercepts: one for subjects and one for items, and a slope for item consistency on participant intercepts. Here and elsewhere, we used the maximal random effect structure that did not cause convergence problems. Linear mixed modelling was implemented using the lme4 (Version 1.1-26; Bates et al., 2015) and lmerTest packages (Version 3.1-3; Kuznetsova et al., 2018). The model formula was ‘response_diversity ~ item_consistency × literacy_skill + (1|item) + (1 + item_consistency|participant)’. Full model outputs for all models can be found on OSF. Here and elsewhere, when the independent variables were centred and an interaction term was present in the model, each main effect should be interpreted as an effect when other variables take their average values.

**Fig. 2:**
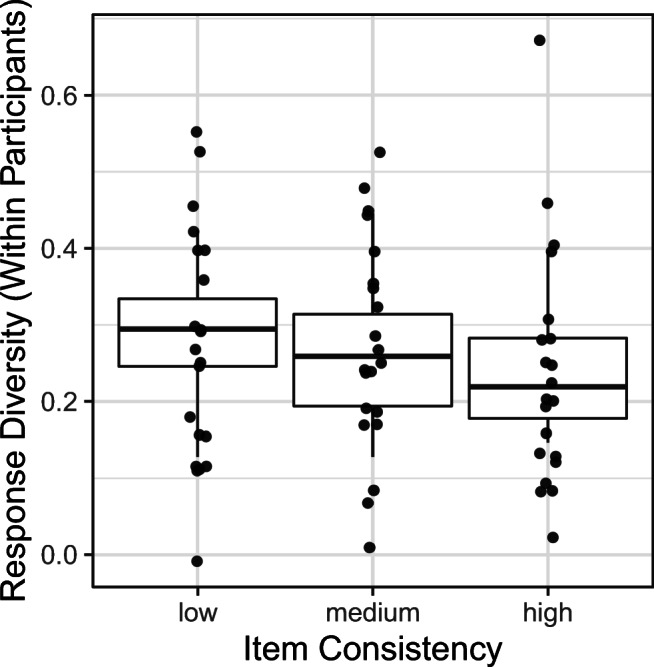
The effect of item consistency on variability of responses within participants (i.e., response diversity; Analysis 1). Each data point corresponds to one participant. The consistency variable was treated as categorical for the purposes of illustration only. The whiskers represent 95% confidence intervals; the boxes span two quartiles (25% and 75%). Higher-consistency items elicit a lower response diversity within participants compared to lower-consistency items

Results indicated a significant main effect of item consistency (*B* = –.048, *SE* = .020, *df* = 42.486, *t* = –2.347, *p* = .024). Nonwords that comprised more inconsistent graphemes yielded greater response diversity than nonwords that comprised more consistent graphemes (see Fig. 2). No other effects reached significance. We calculated coefficients of determination (i.e., *R*^2^ for the entire model) using the r.squaredGLMM function from the MuMIn package (Version 1.43.17; Barton, 2009). The conditional *R*^2^ of the entire model was 17%, while the marginal *R*^2^ indicating how much variance was explained by fixed effects was 2%. These 2% were explained by item consistency.

## Analysis 3: How does session influence intrasubject variability?

In this section, we take advantage of trial-level mixed modelling and develop a new DV, novel pronunciation use, in order to investigate the dynamics of pronunciation change from session to session. Each pronunciation in Sessions 2, 3, 4, and 5 was coded as novel (1; i.e., not used by this participant for this nonword in any previous session) versus old (0; i.e., used previously). For example, the responses of participant AF16 for nonword item BLISPLE were identical across sessions. Therefore, they were coded as 0, 0, 0, 0 (in Sessions 2 to 5, respectively). This participant’s responses to BEBEL were coded as 1, 0, 0, 0, as this participant changed their response from /bɛbəl/ to /bɛbɛl/ in Session 2. Note that novel pronunciations could arise in late sessions without any changes in prior sessions. This was the case for participant AH21, whose response for DEBOME changed in Session 4 (to /dɛbɒm/) from the pronunciation they had been using earlier in Sessions 1–3 (/dɛbəʊm/).

We used generalised mixed effects modelling for a binomial outcome. The model of novel pronunciation use included three predictors (item consistency, literacy skill, and session number) and all their two-way interactions, as well as two random intercepts, one for subjects and one for items. Session was a numeric variable. All variables were centred. The model formula was ‘novel_pronunciation ~ session × item_consistency + session × literacy_skill + item_consistency × literacy_skill + (1|participant) + (1|item)’.

**Fig. 3:**
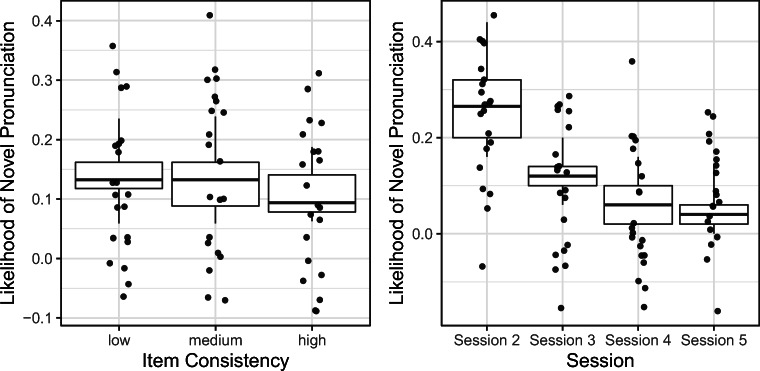
Effects of item consistency and session on the likelihood of a novel pronunciation. Each data point corresponds to one participant. Figures are based on actual observations. The whiskers represent 95% confidence intervals; the boxes span two quartiles (25% and 75%). Left-hand panel: High-consistency items elicit fewer novel pronunciations compared with low-consistency items. Right-hand panel: Novel pronunciation occur in early sessions more often than in late sessions

Results indicated a significant main effect of session (*B* = –.841, *SE* = .057, *z* = –14.653, *p* < .001) and a significant main effect of item consistency (*B* = –.21, *SE* = .103, *z* = –2.046, *p* = .041). These effects are illustrated in Fig. 3. Nonwords with low item consistency that consisted of unpredictable graphemes generated novel pronunciations more often. Further, the likelihood of novel nonword pronunciations was higher in earlier sessions than in later sessions. No other effects reached significance. The conditional *R*^2^ of the entire model was 27%, while the marginal *R*^2^ indicating how much variance was explained by fixed effects was 17% (theoretical values; the corresponding delta values were 12% and 8%, respectively). Further, we inferred the amount of variance accounted for by each significant fixed effect using the partR2 package (Version 0.9.1; Stoffel et al., 2020). The fixed effect of session explained 6.9% of variance and the effect of item consistency accounted for 0.5% of variance.

## Analysis 4: How does repeated testing influence variability across individuals?

The finding that session order negatively affected the likelihood of a novel response indicated that participants were gradually “settling” on one of their prior responses. The question is, were all participants settling on the same response for each nonword, or were they settling on different responses? If so, we would expect that differences across participants that we observed in early sessions would simply propagate into later sessions.

We characterised the extent to which each participant’s response set differs from the response set of every other participant within each session. Response set refers to all responses given by a participant to 50 nonwords in a given session. Note that the final session data from two participants was not available, therefore we had 108 response sets in total. We constructed five (number of participants) × (number of nonwords) matrices and calculated distances between every two rows within each matrix. The difference between two response sets was expressed in terms of Gower distances using the *daisy* function from the R package *cluster* (Version 2.1.0; Maechler et al., 2019). The total number of distances was 1,114 (231 two-set combinations for Sessions 1–4 and 190 two-set combinations for Session 5).

**Fig. 4:**
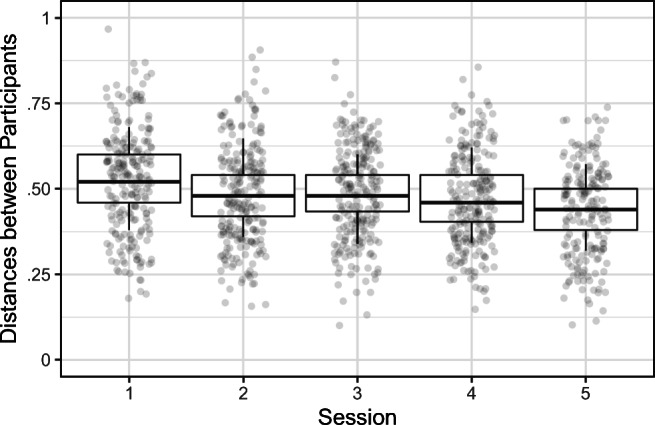
Participants gradually become more similar to each other in terms of how they read nonwords across sessions. The whiskers represent 95% confidence intervals; the boxes span two quartiles (25% and 75%)

The distance values served as a DV in a linear mixed model that included with session number (scaled) and two random intercepts, one for each participant in the pair. Random slopes for the effect of session were also included. The model formula was ‘distance ~ session + (1 + session|participant_1_) + (1 + session|participant_2_)’. The effect of session was significant (*B* = –.022, *SE* = .004, *df* = 28.071, *t* = –5.344, *p* < .001), such that distances between every two participants decreased through Sessions 1 to 5. Our model explained 68% of variance with 5.6% of variance explained by the fixed effect. These results are illustrated in Fig. 4.

## Analysis 5: How does repeated testing influence participants’ pronunciations?

Our Analysis 4 indicated that participants may have abandoned some pronunciations for at least some nonwords. This prompted a further question: Which pronunciations were more likely to be pruned?

Our DV, pronunciation frequency, indicated how many participants used a pronunciation in a given session. For example, pronunciation /eɪdæs/ was used for ADASE twice (i.e., by two participants) in Session 1. Pronunciation frequency ranged from 0 (i.e., not used by anyone in a given session) to 22 (used by everybody in a given session). We excluded Session 5 from this analysis because fewer participants took part in it, and this could bias our DV and our results. The variable was centred. In order to identify pronunciations whose frequencies decreased, we fitted a linear model ‘pronunciation_frequency ~ 1 + (1 + session|pronunciation)’ and extracted the intercepts and the slopes associated with the random effect. The intercepts characterised relative differences in pronunciation frequency among pronunciations, while the slopes characterised the extent of session-to-session change in their frequency of use. For example, /əðeɪs/ was characterised by the intercept of –0.42 (indicating that this pronunciation was among the least frequent; intercepts ranged between –0.56 and 3.79) and the slope of –.03 (indicating that its frequency of use decreased over time; slopes ranged between –.15 and .21). The correlation between intercepts and slopes was *r* = .52 (*N* = 386, *p* < .001) suggesting that more frequent pronunciations tended to spread across participants over time, while less frequent pronunciations tended to drop out of use.

## General discussion

We asked skilled adult readers to read aloud the same set of 50 English nonwords on five occasions. We found that participants did not pronounce these in the same way from session to session. Contrary to our prediction, we found no evidence that the measures of literacy, as indexed by vocabulary and spelling tests, explained any variability across or within individuals. It is likely that our study with 22 participants was underpowered for explaining participant-based variation. Nonetheless, substantial variation both within and across individuals clearly exists. Our view is that a good understanding of how and why nonword reading varies within individuals is essential for understanding variability across individuals and issues surrounding the formation and exploitation of spelling–sound knowledge more generally.

Readers showed greater session-to-session variability in their responses and were more likely to come up with a novel pronunciation on a testing occasion when nonwords consisted of graphemes that could be pronounced in multiple ways across English words. This effect is likely to arise because individuals’ spelling–sound knowledge is probabilistic in nature. In line with this, readers’ behaviour has been shown to mirror variations in the linguistic environments to which they are exposed (Siegelman et al., 2020; Ulicheva et al., 2020). It is not surprising then that when such variations are present (as for inconsistent patterns), individuals show increased variation from session to session.

Our study has implications for interpreting snapshot studies of nonword reading. We found evidence that pronunciation variability was not uniformly distributed across sessions. Novel pronunciations were less likely in later sessions (Analysis 3), and participants became more similar to each other as the sessions progressed (Analysis 4), suggesting that some pronunciations were selectively pruned. Further examination suggested that infrequent pronunciations were those most likely to decrease in frequency across sessions (Analysis 5). This implies that these pronunciations may not reflect individuals’ underlying spelling–sound knowledge. This interpretation would suggest that repeated testing reduces random variation across individuals, leaving us with differences more strongly related to underlying knowledge. Therefore, one implication of this work is that when the nonword reading task is used on a single occasion, infrequent pronunciations may not be characteristic of long-term spelling–sound knowledge and should be analysed and interpreted with caution.

The second implication of this work has to do with how participant-level and item-level effects on variability in snapshot-based studies are interpreted. For instance, an effect of spelling–sound consistency has been reported on pronunciation variability across individuals in snapshot-based studies (Siegelman et al., 2020; Steacy et al., 2019; Treiman & Kessler, 2006; see also Mousikou et al., 2017). In these studies, the effect of pattern inconsistency on variability in nonword pronunciations across participants has been ascribed to differences in individuals’ spelling–sound knowledge. On the other hand, we reported here that inconsistent patterns also elicit more intrasubject variability. This prompts the question: Does the variation in responses to inconsistent patterns observed across individuals truly stem from differences in their long-term knowledge, as has been suggested? Our findings suggest that at least some of the variation that has been previously interpreted as reflecting differences across individuals may be explained by stochastic processes that occur within individuals. Thus, researchers should evaluate the possibility that differences across individuals observed on a single occasion could be induced by processes that occur at the level of a single individual. In order to make progress in interpreting differences arising across individuals, it is essential to develop techniques for isolating true differences across participants from those that stem from intraindividual variation.

One way to advance our understanding of variability in nonword reading involves computational modelling. This requires the development of specific testable hypotheses regarding the mechanisms that promote and constrain session-to-session variability. For example, variation from session to session might emerge if noise is present in the reading system (e.g., the amount of phonological noise in the PDP models; Rueckl et al., 2019). This variation could be constrained by prior exposure to certain pronunciations or even people’s recent experiences with words (cf. Rodd et al., 2016).

In sum, we observed that a single individual may vary in how they pronounce nonwords across occasions. We have argued that understanding session-to-session variability is essential for interpreting variability across individuals that has been documented in single-snapshot studies of nonword reading aloud. Furthermore, we have shown that intersession variability is an interesting phenomenon in its own right and may be able to shed light of the form of spelling–sound knowledge that people possess and exploit on a given occasion.

## Appendix

Fifty nonwords used in the multisession experiment. These nonwords were selected from Mousikou et al. (2017), so that they vary in terms of how many different responses they elicited across participants in that study (entropy values for selected nonwords ranged from 0.38 to 2.35). The histogram suggests a normal distribution of across-participant entropy values.

abast, adase, arroste, bairtul, bastle, bebel, bedit, beglets, blisple, budgord, byfane, clisple, confant, conglire, debome, debort, drafforn, enmil, esude, evove, exbrafe, finklood, flactuse, forsose, glorak, hanep, hanslood, huckep, imbroths, ingote, ingrap, jemsim, jubtle, juckem, miscleaf, muntod, nasple, outslom, pasake, pazim, practash, rebuse, reote, shigack, stramble, suldend, tactond, tactosh, turlang, unbove.

## Footnotes

1 This decision is not meant to reflect any theoretical assumption regarding syllable identification being an early stage of word processing in all individuals. This was done for purely practical reasons (i.e., to increase the number of words from which grapheme-to-phoneme correspondences can be reliably extracted).

2 We are unaware of any standard means of calculating the consistency of a polysyllabic word, although typically consistency for monosyllables is an average of the consistency of all grapheme constituents (Mousikou et al., 2017).

3 Though we were not interested in response times, we included an instruction that emphasised speed because this is relatively standard in experiments involving nonword reading (e.g., Pritchard et al., 2012). It is possible that responses are more variable when instructions emphasise speed, although it is also possible that greater variability should arise when participants have more time to reflect on their responses.
